# Insensibility to Pain by Inhalation of the Vapor of Sulphuric Ether

**Published:** 1847-01

**Authors:** 


### EDITORIAL DEPARTMENT.

---

*Insensibility to pain by Inhalation of the Vapor of Sulphuric Ether.*—This is the all engrossing medical topic at the present moment, and bids fair so to continue for some time to come. The object which the new method professes to effect is of such importance, not alone to the surgeon, but to the patient, that it must necessarily excite interest, both with the profession and the public. Experiments sufficiently numerous and authentic, have been already communicated to settle the question as to the fact that insensibility may be produced in a large proportion of cases. It remains to be determined whether the process is attended with any liability to dangerous consequences, which should preclude its general adoption in surgical operations. To settle this point, further observations are requisite, and until this point is settled, it is prudent to refrain from indulging enthusiastic expectations of its availability. It does not, as yet, appear that any serious evils have resulted from its application, although in several instances patients have experienced more or less unpleasant effects of temporary duration. We cannot but fear that cases will occasionally occur in which these effects will assume so grave a character as to induce too much apprehension on the part of the operator, and the patient, to resort to it indiscriminately, Should this happen, however, it does not follow that it is to be abandoned. The true course will then be, to endeavor to ascertain the circumstances which render it appropriate and free from danger, in order to be guided by these circumstances in its application.

The rationale of the operation of the ethereal vapor in producing a state of temporary insensibility is an interesting topic of inquiry. We assume that the vapor is simply that of sulphuric ether. This we understand from good authority is the fact, and is not denied by those who are interested in the patented discovery. The inhalation of this vapor is not a matter of novelty. For the sake of amusement it has often been administered, occasioning, under these circumstances, a species of intoxication, accompanied by agreeable hilarity. In order to produce a passive condition of insensibility it requires to be inhaled in larger quantities, and with the moral influences derived from the expectation that a painful operation is to be performed. The latter doubtless exerts no inconsiderable effect in giving the phenomena the direction to be desired. The same person inhaling the same amount of the vapor for the purpose of amusement at one time, and at another time, for the sake of deadening sensibility, will probably in the one case be boisterous and unruly, and in the other lethargic and entirely manageable. It seems most rational to suppose that the physiological action is exerted primarily on the blood. How much is due to the mere suspension of proper oxygenation, and how much to peculiar modifications by the combinations of the vapor remain to be determined.

The importance of this discovery, should its supposed advantages be fully realized, not alone consists in divesting surgery of suffering. Every surgeon is aware, that in connection with pain and mental apprehension, the system receives a shock, which is often a source of serious injury, and may even, in itself, prove fatal to the patient. There seems good ground for supposing with Dr. Pierson, that the evils arising from these causes may in a measure be prevented, and the usual constitutional irritation greatly mitigated, provided a state of insensibility can be safely maintained during the performance of surgical operations. Should this supposition be verified, the benefits conferred on operative surgery and humanity by the application of this Letheon agent, will constitute a memorable era in the history of medicine.

There is one point connected with this subject to which we cannot refer with any satisfaction. We allude to the attempt to secure an exclusive proprietorship by virtue of a patent. We perceive that this has excited considerable discussion and feeling among the profession at Boston; and as we think justly—or, rather, there is hardly room for discussion, but only for an animadversion. There can be no question as to the professional merits of the matter. It is in direct, palpable opposition to vital principles of medical ethics, by which, as a profession, we declare ourselves to be governed.

Of the alledged proprietor of the vapor we know nothing. He may not be a member of the medical profession, and therefore not consider himself holden by its rules. If so, it is a matter between himself and his own conscientious views of propriety and morality. But the reputed discoverer is well known as a gentleman of eminent scientific attainments, whose reputation has conferred honor on the medical profession of this country. We regret to see it intimated that he is in any way compromised by the effort to keep the preparation secret, or to limit its usefulness. How can we consistently maintain the position we have assumed toward the thousand secret remedies which are hawked about the country, when we are ourselves open to the imputation that the same course is pursued by members, and distinguished members of our profession! Unless every attempt of this kind be met with a prompt and emphatic repudiation by the profession, it is in vain to disclaim relationship with inventors of patented nostrums, and the term *quackery* may as well be dropped from our vocabulary. It is not to be denied that every valuable discovery or invention in medicine, as in other departments of useful science and art, is deserving of reward. It would be but fair that the inventor or discoverer should receive a pecuniary equivalent for the benefits which he confers upon society. How this renumeration is to be effected, or whether it be practicable in all cases we will not stop to inquire. This personal consideration, it is manifest, should weigh nothing in comparison with what is due to humanity, to the dignity, honor and usefulness of the medical profession, as well as to the obligations of compact which membership with the profession imposes—but we are pursuing a train of remark which is superfluous, because it is universally known, and acknowledged to be true. We would repeat the expression of regret, that this discovery has not been, ere this, formally communicated by the discoverer to the profession; and we sincely trust, both for the credit of the profession, and for the professional character of the discoverer, for whom we entertain the highest respect, that the matter will not be permitted to remain in its present position.

The legal efficacy of a patent in this case we learn is exceedingly questionable. It is a simple, well known medical agent, and is administered in the same way essentially as heretofore for amusement. The discovery consists in the peculiar effects produced by its administration in view of a surgical operation. We do not say this to depreciate the merit of the discovery, for we consider this not less than if some new and intricate compound had been ascertained, or some peculiar method of administration contrived. The procedure in vaccination was a simple affair, but not on that account less useful or brilliant as a discovery. But in view of the circumstances mentioned, it is rational to doubt if it can be legally appropriated for the exclusive benefit of any limited number of individuals. Assuming for the moment that it could be demonstrated that Hydropathy will cure all diseases, could the use of water be patented! At all events we understand that the Dentists and Surgeons in Boston and its vicinity have no hesitation in employing the ethereal vapor without leave or license from any one; and we presume their example will be followed throughout the country. Before the appearance of our next number we shall probably have opportunities to witness repeated instances of its exhibition; and in resuming the subject, we will then describe the method to be pursued, so that those of our medical readers who do not receive the necessary information from other sources may be prepared to avail themselves of it should they wish to do so.

